# A comparison of the clinical outcomes of esophagectomy and chemoradiotherapy after noncurative endoscopic submucosal dissection for esophageal squamous cell carcinoma

**DOI:** 10.1007/s00595-018-1650-y

**Published:** 2018-03-12

**Authors:** Yasufumi Koterazawa, Tetsu Nakamura, Taro Oshikiri, Shingo Kanaji, Shinwa Tanaka, Tsukasa Ishida, Kimihiro Yamashita, Takeru Matsuda, Yoshinori Morita, Satoshi Suzuki, Yoshihiro Kakeji

**Affiliations:** 1 Division of Gastrointestinal Surgery, Department of Surgery, Kobe University Graduate School of Medicine, 7-5-2, Kusunoki-cho, Chuo-ku, Kobe, Hyogo 650-0017 Japan; 2 Division of Gastroenterology, Department of Inter Medicine, Kobe University Graduate School of Medicine, Kobe, Hyogo Japan

**Keywords:** Superficial esophageal cancer, ESD, Esophagectomy

## Abstract

**Purpose:**

Endoscopic submucosal dissection (ESD) is widely used to treat esophageal cancer, but some patients require additional treatment due to the possibility of lymph node metastasis. The aim of this study was to elucidate the clinical outcomes of these additional treatments.

**Methods:**

The study included 59 patients who developed superficial esophageal squamous cell carcinoma after noncurative ESD treated between 2005 and 2016, of whom 28 underwent esophagectomy and 31 received chemoradiotherapy (CRT).

**Results:**

The median follow-up periods were 45 months in the esophagectomy group and 41 months in the CRT group. The overall survival did not differ significantly between the groups (*P* = 0.46). However, there were no recurrences in the esophagectomy group, and the disease-specific survival rate was significantly higher in this group (*P* = 0.042). Among the patients at high risk for recurrence due to massive tumor invasion (≥ SM2) with lymphovascular invasion (esophagectomy group, six patients; CRT group, ten patients), none in the esophagectomy group had recurrence, whereas four in the CRT group died of esophageal cancer (*P* = 0.031).

**Conclusion:**

The overall survival did not differ significantly between the groups. However, compared with CRT, esophagectomy provided more favorable disease control for patients with massive tumor invasion (≥ SM2) with lymphovascular invasion.

## Introduction

Esophageal cancer is the sixth-most common cause of cancer-related mortality worldwide [1]. Most cases of esophageal carcinoma in Japan are squamous cell carcinoma (SCC) [2]. The etiology of esophageal SCC differs from that of adenocarcinoma. Alcohol consumption and smoking are risk factors for esophageal SCC, and in East Asia, many individuals have a flushing response after alcohol intake, which increases the risk of esophageal SCC [3]. This flushing is due to the presence of inactive aldehyde dehydrogenase-2. Therefore, in East Asia, SCC is diagnosed more frequently than adenocarcinoma. The advancement of diagnostic technology has improved the detection rate of superficial esophageal carcinoma [4]. Endoscopic submucosal dissection (ESD) can completely remove diseased lesions via dissection through the submucosa (SM), and it is now widely used to treat superficial esophageal cancer in Japan [5].

A correlation exists between the rate of lymph node metastasis and the depth of tumor invasion of superficial esophageal cancer. The reported rates of lymph node metastasis are 0–4.0% for cancers in the epithelium (EP) and lamina propria mucosae (LPM), 0–15% in the muscularis mucosae (MM), and > 20% in the SM [6–9]. Yamashita et al. [9] showed that the incidence of metastasis in mucosal cancer was associated with lymphovascular invasion and reported cumulative 5-year metastasis rates of 46.7 and 0.7% in patients with mucosal cancer with and without lymphovascular invasion, respectively. Therefore, additional definitive treatment is key for patients who have tumors invading the SM and MM and lymphovascular invasion, even if no lymph node metastasis is clinically observed.

The primary additional treatment after noncurative ESD is esophagectomy, which gives favorable disease control for superficial esophageal carcinoma [4, 10]. However, there is also increasing evidence supporting the efficacy of chemoradiotherapy (CRT) for superficial esophageal SCC [11, 12]. Patients who cannot undergo esophagectomy or who choose CRT generally receive CRT as additional treatment [12], but no studies have compared esophagectomy and CRT after noncurative ESD.

Therefore, the aims of this study were to elucidate the efficacy and clinical outcomes of esophagectomy and CRT, analyze the adverse effects of these additional treatments, and estimate the extent of lymph node metastasis in surgical specimens.

## Patients and methods

Endoscopic submucosal dissection (ESD) was performed for patients with esophageal cancer clinically diagnosed as T1a (EP, LPM, or MM, including inaccurately diagnosed MM or SM1) according to the guidelines established by the Japan Esophageal Society [14]. The diagnosis of tumor invasion was performed with high-resolution endoscopy with iodine staining, magnified endoscopy with narrow-band imaging, and endoscopic ultrasonography. Dissected specimens were examined by at least two experienced pathologists after ESD to determine the histological type, depth of invasion, lateral and vertical resection margins, and presence of lymphovascular invasion.

Additional definitive treatment was recommended for patients who had undergone noncurative ESD for SM cancers or MM cancers with lymphovascular invasion and a positive resection margin [8, 9, 13, 14]. A total of 940 patients with superficial esophageal carcinoma underwent ESD between 2005 and 2016. Among them, we selected 59 patients, of whom 28 (47%) underwent esophagectomy and 31 (53%) received CRT by choice or because their condition did not permit surgery.

Esophagectomy was performed via thoracoscopic or right thoracotomy resection with lymph node dissection. The pathological stage was determined according to the eighth edition of the tumor–node–metastasis classification established by the Union for International Cancer Control. CRT was performed using a protocol similar to that of the Japan Clinical Oncology Group Study JCOG0508 protocol [14] as follows: Cisplatin was administered at a dose of 70 mg/m^2^ via slow-drip infusion on days 1 and 29, fluorouracil was administered at a dose of 700 mg/m^2^ per day via continuous infusion for 24 h on days 1–4 and 29–32, and the dose of radiotherapy was 41.4 Gy for patients with negative endoscopic resection margins and 50.4 Gy with boost at the primary site for patients with positive resection margins.

Patients’ data, such as their age, sex, and comorbidities, were collected from medical records. Each patient’s condition was assigned an American Society of Anesthesiologists physical status (ASA PS) classification score. Pathological data after ESD and the clinical outcomes after each additional treatment were recorded, and survival curves were drawn using the Kaplan–Meier method and analyzed with the log-rank test. All analyses were performed with the JMP^®^ software program, ver. 10 (SAS Institute Inc., Cary, NC, USA), and *P* values of < 0.05 were considered significant. The study protocol was approved by the Ethics Committee of Kobe University Hospital and conformed to the provisions of the 1995 Declaration of Helsinki (as revised in Edinburgh in 2000). All study participants gave their informed consent, and patient anonymity has been preserved.

## Results

### Clinical features and pathological findings after ESD

The clinical features and pathological findings in 59 patients who underwent esophagectomy or CRT are shown in Table 1. The 28 patients in the esophagectomy group (24 men and 4 women) had a median age of 66 years (range 47–77), and the 31 patients in the CRT group (24 men and 7 women) had a median age of 68 years (range 50–81). There were no significant differences in ASA PS classification, comorbidities, or tumor location or size between the groups. The reasons for additional treatment (multiple reasons in some cases) were submucosal invasion (*n* = 20, 71%), lymphatic invasion (*n* = 16, 57%), vascular invasion (*n* = 1, 3.6%), positive horizontal margins (*n* = 1, 3.6%), and positive vertical margins (*n* = 3, 10.7%) in the esophagectomy group and submucosal invasion (*n* = 28, 90%), lymphatic invasion (*n* = 11, 35%), vascular invasion (*n* = 3, 9.7%), positive horizontal margins (*n* = 2, 6.5%), and positive vertical margins (*n* = 4, 12.9%) in the CRT group. The reasons for additional treatment did not differ significantly between the groups.

**Table 1:** Clinical features and pathological findings after endoscopic submucosal dissection

	Esophagectomy group (*n* = 28)	CRT group (*n* = 31)	*P* value
Median age, years (range)	66 (47–77)	68 (50–81)	0.16
Sex (%)			0.41
Male	24 (85.7%)	24 (77.4%)	
Female	4 (14.3%)	7 (22.6%)	
ASA PS classification			
1	15 (53.6%)	16 (51.6%)	0.88
2	11 (39.3%)	8 (25.8%)	0.27
3	2 (7.1%)	7 (22.6%)	0.09
Common comorbidities			
Hypertension	8 (28.5%)	12 (38.7%)	0.41
Diabetes mellitus	2 (7.1%)	4(12.9%)	0.46
Cardiovascular disease	0 (0%)	1 (3.2%)	0.25
Location of tumor			0.56
Ce/Ut/Mt/Lt/Ae	0/3/20/4/1	2/4/20/5/0	
Tumor size, mm (range)	34 mm (9–100)	29 mm (7–80)	0.37
Depth of tumor invasion			0.07
T1a	8 (29%)	3 (10%)	
T1b	20 (71%)	28 (90%)	
Lymphatic invasion			0.71
Positive	16 (57%)	11 (35%)	
Negative	12 (43%)	20 (65%)	
Venous invasion			0.33
Positive	1 (3.6%)	3 (9.7%)	
Negative	27 (96.4%)	28 (90.3%)	
Horizontal margin			0.61
Positive	1 (3.6%)	2 (6.5%)	
Negative	27 (96.4%)	29 (93.5%)	
Vertical margin			0.79
Positive	3 (10.7%)	4 (12.9%)	
Negative	28 (90.3%)	27 (87.1%)	

*CRT* chemoradiotherapy, *ASA PS* American Society of Anesthesiologists physical status

Compared with the esophagectomy group, the CRT group had more patients with submucosal invasion, but this difference did not reach significance (*P* = 0.07). The number of patients who received additional treatment for other reasons (lymphatic invasion, vascular invasion, positive horizontal margins, and vertical margins) also did not differ significantly between the groups (*P* = 0.71, *P* = 0.33, *P* = 0.61, and *P* = 0.79, respectively).

### Clinical outcomes in the treatment groups

**Fig. 1:**
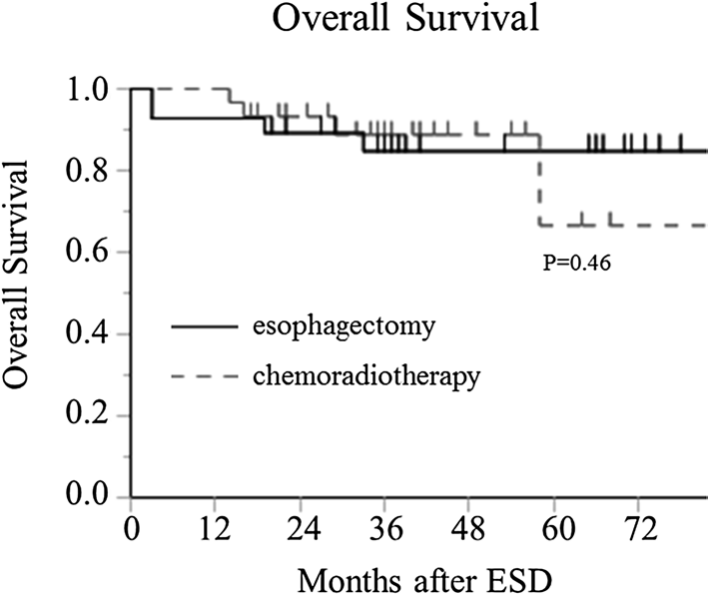
There was no marked difference in the overall survival between the esophagectomy group (solid line) and the chemoradiotherapy (CRT) group (dotted line)

The median follow-up periods were 45 months (range 3–89 months) in the esophagectomy group and 41 months (range 12–84 months) in the CRT group. Ten patients died during the observation period, but no significant difference in the overall survival was found between the groups (*P* = 0.46; Fig. 1). During follow-up, there was no recurrence of esophageal cancer in the esophagectomy group, but five patients had recurrence in the CRT group (Table 2), including abdominal lymph node recurrence in two patients, and mediastinal lymph node, liver metastasis, and local recurrence located at the same site after ESD in one patient each (see Table 2). Three of these recurrences (one abdominal lymph node recurrence and the mediastinal lymph node recurrence and local recurrence) were inside the radiation field, and the other two (one abdominal lymph nodal recurrence and the liver metastasis recurrence) were outside the radiation field.

**Table 2:** Clinical and pathological characteristics and outcomes of patients with recurrent cancers

Age (years)/sex	Tumor depth	Tumor location	Lymphovascular invasion	Resection margin (horizontal or vertical)	RT (Gy)	Time to recurrence (months after ESD)	Recurrence site	Inside or outside radiation field	Outcome
73/male	SM2	Mt	Ly+V−	Vertical positive	50.4	17	Abdominal LN	Inside	Death
69/male	SM2	Mt	Ly+V−	Vertical positive	50.4	12	Abdominal LN	Outside	Death
79/male	SM2	Mt	Ly+V−	Negative	41.4	43	Liver metastasis	Outside	Death
50/male	SM2	Mt	Ly+V−	Negative	41.4	51	Mediastinal LN	Inside	Death
63/male	SM1	Mt	Ly−V−	Negative	41.4	24	Post-ESD scar	Inside	Alive

*LN* lymph node, *RT* radiotherapy, *SM* submucosa, *ESD* endoscopic submucosal dissection

**Fig. 2:**
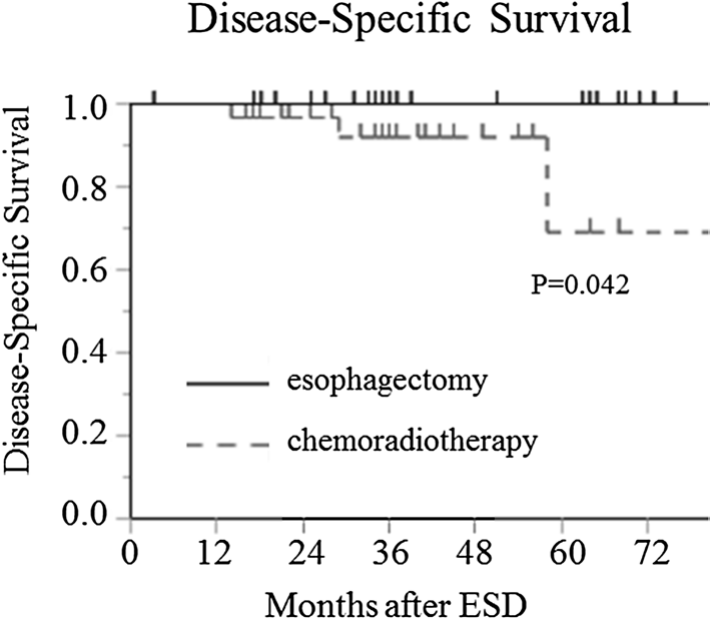
Compared with that in the CRT group (dotted line), the disease-specific survival rate in the esophagectomy group (solid line) was significantly higher

Four patients died of esophageal cancer in the CRT group. These patients were at high risk for recurrence due to massive tumor invasion (≥ SM2) with lymphovascular invasion. In total, ten patients in the CRT group and six in the esophagectomy group were considered high risk (*P* = 0.34), but all high-risk patients in the esophagectomy group are alive without recurrence at the time of this writing (*P* = 0.031; Table 3). Therefore, the disease-specific survival rate was significantly lower in the esophagectomy group than in the CRT group (*P* = 0.042; Fig. 2).

**Table 3:** Patients at high risk for recurrence due to ≥ SM2 cancer with lymphovascular invasion

	Esophagectomy (*n* = 28)	CRT (*n* = 31)	*P* value
Patients at high risk for recurrence (%)	6 (21.4%)	10 (32.3%)	0.34
Patients with recurrence/patients at high risk for recurrence (%)	0/6 (0%)	4/10 (40%)	0.031

*CRT* chemoradiotherapy, *SM* submucosa

### Clinicopathological features after esophagectomy

The tumor–node–metastasis pathological stages of patients in the esophagectomy group were pStage IA (*n* = 7, 25.0%), IB (*n* = 12, 42.9%), and IIB (*n* = 9, 32.1%). Eleven patients (39.3%) had multiple lesions, and in two of these patients, the second lesion was not detected before esophagectomy (Table 4). Pathological lymph node metastasis was noted in nine patients (32.1%), whose clinical diagnosis was negative for metastasis (Table 5). Seven patients had a single lymph node metastasis in the cervix, mediastinum, or abdomen, and two patients had two lymph node metastases in the mediastinum. In case 9, a high-risk patient (SM2 with lymphovascular invasion), the tumor location was the lower thoracic esophagus, but pathological lymph node metastasis occurred at the upper thoracic paraesophageal lymph node.

**Table 4:** Clinicopathological features after esophagectomy (*n* = 28)

Item	Value
Pathological tumor depth	
T1a	8 (29%)
T1b	20 (71%)
Numbers of tumors	
Single	17 (60.7%)
Multiple	11 (39.3%)
Lymph node metastasis	
Positive	9 (32.1%)
Negative	19 (67.9%)
Pathological stage	
Stage IA	7 (25.0%)
Stage IB	12 (42.9%)
Stage IIB	9 (32.1%)
Approach	
Thoracoscopic esophagectomy	25 (89.2%)
Right thoracotomy	3 (10.7%)
Clinical outcomes	
Recurrence	0 (0%)
Cancer death	0 (0%)

Determinations of the pathological depth of tumor invasion, lymph node metastasis, and pathological findings were based on the eighth edition of the Union for International Cancer Control tumor–node–metastasis classification of malignant tumors

**Table 5:** Features of nine patients with pathological lymph node metastasis

Case	Age (years)/sex	Tumor location	Tumor depth	Lymphovascular invasions	Tumor size (mm)	Lymph node metastasis	Number of metastatic lymph nodes
1	72/male	Mt	MM	Ly + V−	100	Cervical (paraesophageal)	1
2	70/male	Mt	SM1	Negative	8	Cervical (paraesophageal)	1
3	66/male	Mt	SM1	Ly + V−	12	Mediastinal (Lt tracheobronchial)	1
4	63/male	Mt	SM1	Ly + V−	54	Mediastinal (middle thoracic paraesophageal)	1
5	66/male	Mt	SM1	Ly + V−	39	Mediastinal (Lt recurrent nerve)	1
6	52/male	Ut	SM2	Negative	26	Mediastinal (Lt recurrent nerve)	2
7	69/male	Mt	SM2	Negative	20	Mediastinal LN (upper thoracic paraesophageal and posterior mediastinal)	2
8	72/male	Mt	SM2	Negative	48	Abdominal (lesser curvature)	1
9	69/male	Lt	SM2	Ly + V−	36	Mediastinal (upper thoracic paraesophageal)	1

*LN* lymph node, *MM* muscularis mucosae, *SM* submucosa

### Adverse effects of esophagectomy and CRT

The effects of the ESD scar and adhesion on postoperative complications were examined by comparing esophagectomy after ESD with conventional esophagectomy. Complications were defined using the Clavien–Dindo classification [15]. Among patients who underwent esophagectomy after ESD, five (17.8%) had anastomotic leakage, eight (28.5%) had pulmonary complications, and three (10.7%) had recurrent nerve palsy. These rates did not differ significantly from the respective rates of 14.6, 21, and 13% after conventional esophagectomy (Table 6).

**Table 6:** Postoperative complications in esophagectomy after ESD and conventional esophagectomy

Item	Esophagectomy after ESD (*n* = 28)	Conventional esophagectomy (*n* = 301)	*P* value
Median age, years (range)	66 (47–77)	68 (44–84)	0.71
Sex (male/female)	23/5	248/53	0.69
Anastomotic leakage	5 (17.8%)	28 (14.6%)	0.65
Pulmonary complication	8 (28.5%)	63 (21%)	0.47
Recurrent nerve palsy	3 (10.7%)	39 (13%)	0.64
Mortality	2 (7.1%)	3 (1.0%)	0.06

*ESD* endoscopic submucosal dissection

The toxicities of CRT are summarized in Table 7. We assessed adverse effects in this study in accordance with the Common Terminology Criteria for Adverse Events, ver. 4.0 [16]. Grade 3 leucopenia occurred in three patients and grade 3 esophagitis in one patient. There were no other grade 3 complications (such as pneumonia, sepsis, and liver failure), no late grade 3 toxicities, and no serious (grade 4) adverse events.

**Table 7:** Acute and late toxicities of patients who received additional chemoradiotherapy (*n* = 31)

Toxicities	Grade		
	2	3	4
Acute			
Hematological (leukocytes)	8	3	0
Esophagitis	4	1	0
Nausea	5	0	0
Creatinine	2	0	0
Radiodermatitis	2	0	0
Late			
Arrhythmia	2	0	0
Hypothyroidism	2	0	0

Assessment of toxicities was performed in accordance with the Common Terminology Criteria for Adverse Events, ver. 4.0 [16]

## Discussion

In this study, cT1a cancer (with no lymph node metastasis) was the initial diagnosis for nine patients (32.1%) in the esophagectomy group; however, positive lymph node metastases were observed in the surgical specimens. Motoyama et al. [17] reported that 29% of patients with a clinical diagnosis of cT1a cancer (lymph node–negative before surgery) were ultimately positive for pathological lymph nodes. This finding shows that accurate clinical diagnoses, including lymph node metastases, are difficult to make. In a retrospective analysis of 22,123 patients with esophageal cancer, Rice et al. [18] found that patients with a clinical diagnosis of superficial cancers had survival rates poorer than expected from equivalent pathological categories, which indicates that clinical and pathological diagnoses do not share prognostic implications. These findings add difficulty to decision making regarding clinical treatments.

Positive lymph node metastasis is a prognostic factor in esophageal cancer, and patients with lymph node metastasis may have unfavorable prognoses [19]. Therefore, the treatment strategy for this disease depends on whether or not lymph node metastasis has occurred. Esophagectomy with lymph node dissection is recommended due to its favorable efficacy in patients who have undergone noncurative ESD [17, 20, 21]. However, CRT for patients with superficial esophageal cancer is also efficacious [11, 12]. Patients who cannot undergo esophagectomy or prefer CRT to surgery generally undergo CRT [12]. To our knowledge, this study is the first to analyze the outcomes of these additional treatments.

Both esophagectomy and CRT after noncurative ESD provide favorable disease control, and we found no significant difference in the overall survival between the treatment groups. However, the disease-specific survival differed significantly between the groups. In the CRT group, four cancer-related deaths occurred during follow-up. Patients with SM2 cancer and lymphovascular invasion have a high rate of recurrence [8, 9, 12], and the four patients who died of esophageal cancer were at high risk for recurrence due to massive tumor invasion (≥ SM2) with lymphovascular invasion. For such high-risk patients, we recommend surgical intervention over CRT as the first-line treatment.

In terms of adverse effects, CRT has an advantage over esophagectomy, because it conserves the esophagus and has no postoperative complications. Compared with patients who undergo other gastroenterological surgeries, those undergoing esophagectomy have higher mortality [22–25]. CRT is less invasive than esophagectomy; therefore, patients should meet rigid indications, including age, physical status, and comorbidity criteria, for additional esophagectomy. Among the 28 patients in the esophagectomy group, two (7.1%) died of surgery-related complications after esophagectomy. One patient died because of gastric tube necrosis, and another died because of acute respiratory distress syndrome. These patients were 69 and 70 years of age, and their ASA PS classifications were 1 and 2, respectively. The patients died irrespective of these indications. The mortality of these patients was not related to the effect of the ESD scar. In the present study, the overall mortality rate was 1.5% (5 of 329), both in the esophagectomy after ESD group and conventional esophagectomy group. This rate compares favorably with the results of other studies that reported mortality rates of approximately 5% (range 4–14%) [22–25]. In addition to the rigid indications, improvements in surgical techniques will further reduce surgery-related complications and improve clinical outcomes.

Akutsu et al. [8] found that patients with EP or LPM cancers did not develop lymph node metastasis, whereas such metastasis occurred in 9% of patients with MM cancers, 16% with SM1 cancers, and > 30% with SM2–3 cancers. Therefore, ESD alone is a definitive treatment for patients with EP or LPM cancers, while additional treatment may be required for those with SM cancers. For patients with MM lesions, the survival benefit of additional treatment remains unknown. Eguchi et al. [7] reported that pT1a-MM cancer with lymphovascular invasion is a high-risk condition for lymph node metastasis, similar to the risk of patients with SM cancers. In the present study, one (14.3%) patient with pT1a-MM cancer with lymphovascular invasion (esophagectomy group, seven patients) had lymph node metastasis (case 1; see Table 5). Along with the histopathology of esophageal cancer, individual factors, such as the age, comorbidities, and physical status, may be important when selecting patients for additional treatment.

This study has several limitations, including the retrospective, single-institution design, and small number of patients. Performing prospective randomized studies is difficult; however, further studies with a larger number of patients enrolled from multiple institutions are needed. Within these limitations, we conclude that balancing individual conditions and risk for recurrence is critical when selecting and performing additional treatments after noncurative ESD. We recommend surgery over CRT for patients at high risk due to massive tumor invasion (≥ SM2) with lymphovascular invasion.
